# Phylogenetic Relationships of Five Phallales Species Based on Mitochondrial Genome Analysis

**DOI:** 10.3390/jof12030207

**Published:** 2026-03-13

**Authors:** Yaping Wang, Dan Li, Guoyu Wang, Zhongyao Guo, Xianyi Wang, Hongmei Liu

**Affiliations:** 1 Engineering Research Center of Medical Biotechnology, School of Biology and Engineering, Guizhou Medical University, Guiyang 561113, China; 2 Engineering Research Center of Health Medicine Biotechnology of Institution of Higher Education of Guizhou Province, Guiyang 561113, China; 3 Key Laboratory of Biology and Medical Engineering, Immune Cells and Antibody Engineering Research Center of Guizhou Province, School of Biology and Engineering, Guizhou Medical University, Guiyang 561113, China; 4 State Key Laboratory of Functions and Applications of Medicinal Plants, Guizhou Medical University, Guiyang 561113, China

**Keywords:** Phallales, mitochondrial genome, phylogenetic analysis, *Lysurus*, *Phallus*

## Abstract

Fungi of the Phallales order are globally distributed and are important in forest ecosystems, and many species have medicinal and edible value. However, despite the rich diversity, the information on this order is limited, and its taxonomic classification remains contentious. In this study, the mitogenomes of five species from the Phallales order were sequenced, assembled, annotated, and compared. All five assembled mitogenomes were circular, ranging in size from 41,465 bp to 99,150 bp. Introns and intergenic regions were the key factors for mitogenome size variation in the Phallales order. The arrangement of 15 protein-coding genes, 2 rRNA genes, and 24 tRNA genes was highly conserved among the Phallales species. The only variation observed was the presence of an additional copy of *trnI*, *trnT*, *trnD*, and *trnF* in some mitogenomes. Specifically, the mitogenomes of *P. rugulosus*, *P. hadriani*, *P. rigidiindusiatus*, and *P. dongsun* had an additional copy of *trnI*, *trnT*, *trnD*, and *trnF*, respectively. A phylogenetic analysis produced well-supported phylogenetic tree, indicating that the mitogenome was an effective molecular marker for inferring evolutionary relationships. The phylogenetic analysis showed that the Phallales and Gomphales species share a closer evolutionary relationship. Our results contribute to a better understanding of the evolutionary dynamics, genetic constitution, and systematic classification of this important fungal community.

## 1. Introduction

The order Phallales, within the phylum Basidiomycota, class Agaricomycetes, and subclass Phallomycetidae, comprises a globally distributed group of fungi that represent a significant and integral component of forest ecosystems. Many Phallales species are edible mushrooms with medicinal properties [1,2,3,4]. Certain species within the Phallales order are recognized to possess significant toxic properties, posing serious threats to human health, such as *Lysurus mokusin* (L. f.) Fr. [5,6]. A notable characteristic of the Phallales fungi is their ability to produce foul or distinctive odors, which serve to attract insects for spore dispersal [7]. The indusium may function as a structure that allows insects to climb up to the gleba, thereby attracting insects that are not primarily responsive to the odors [8]. Although stinkhorn mushrooms possess distinctive biological features, they exhibit limited morphological traits suitable for precise taxonomic delineation. Traditionally, taxa possessing a well-developed indusium were assigned to *Dictyophora*, while those lacking this structure were classified under *Phallus*. However, Kreisel found no fundamental morphological distinction between the genera, as a vestigial indusium occurs within the pileus of some *Phallus* species [9]. Consequently, based on the evidence from modern molecular systematics, all former *Dictyophora* species have been incorporated into the genus *Phallus* [10,11]. Morphology-based taxonomic studies present significant challenges for the accurate identification of species within the Phallales order, mainly because most studies on the Phallales fungi rely on field-collected samples. Primordia obtained from different locations and at varying developmental stages often show significant heterogeneity, which is further exacerbated by different environmental factors under natural conditions. Moreover, the rapid elongation of the fruiting body, along with the easily desiccated and fragmented indusium during expansion, substantially complicates accurate field identification [12]. These factors collectively lead to frequent misidentification and nomenclatural errors in taxonomic practice. Several molecular markers, including internal transcribed spacer (*ITS*), large subunit ribosomal RNA gene (*LSU*), RNA polymerase II second largest subunit (*RPB2*), and mitochondrial ATPase subunit 6 (*atp6*), have been used to reconstruct the phylogenetic relationships within the Phallales order [4,13,14]. However, given the vast genetic diversity found in the Phallales order, these sequences provide limited genetic information for inferring relationships among taxa. In fact, the mitochondrial genomes can provide valuable insights into population and evolutionary studies [15,16].

Fungal mitochondrial genomes (mitogenomes) typically comprise a conserved set of core functional genes essential for aerobic respiration and mitochondrial translation. These include 14 protein-coding genes (PCGs), encoding key subunits of the oxidative phosphorylation complexes: NADH dehydrogenase (*nad1*-*nad6*, *nad4L*), cytochrome c oxidase (*cox1*-*cox3*), cytochrome b (*cob*), and ATP synthase (*atp6*, *atp8*, *atp9*). The conserved complement of fungal mitogenomes also includes two ribosomal RNA genes (*rnl* and *rns*, encoding the large and small rRNA subunits, respectively), one ribosomal protein gene (*rps3*), and a suite of approximately 22–26 tRNA genes (*trn*) sufficient for protein synthesis within the organelle [16]. The gene complement of the fungal mitogenomes is relatively conserved, yet the genome size, as well as gene order, exhibits high variability [17]. Studies have shown that the mitogenome serves as a widely utilized molecular marker, characterized by an elevated evolutionary rate and more conserved transcriptional profiles compared to nuclear genes, rendering it a valuable tool for phylogenetic investigations in fungi [18,19,20,21,22,23]. Although high-throughput sequencing has enabled large-scale mitogenome analysis, the mitochondrial genomes of fungi have received less research attention than animals. In particular, the availability of determined Phallales mitogenomes remains insufficient to meet the research demands. Current records in the NCBI database show that mitochondrial DNA sequences have been deposited for only three species belonging to the Phallales order: *Phallus echinovolvatus* [18], *Phallus indusiatus* [18], and *Phallus dongsun* (GenBank accession number: PP621566.1). Of these, the mitogenome of *P. dongsun* lacks comprehensive analysis. Therefore, generating additional mitogenomic information is essential for robust inferences regarding the taxonomic classifications and evolutionary characteristics of the Phallales species.

In this study, five Phallales species collected from Guizhou, Yunnan, and Fujian Provinces, China, including *Lysurus mokusin*, *Phallus indusiatus*, *Phallus hadriani*, *Phallus rigidiindusiatus*, and *Phallus rugulosus*, were sequenced and assembled. This investigation aimed to: (1) characterize and analyze the mitogenome of five Phallales species and (2) elucidate the evolutionary relationships within Agaricomycetes using complete mitogenomic sequences. The generated mitogenomes provide a foundational genomic dataset for elucidating structural features, facilitating deeper understanding of phylogenetic relationships and evolutionary patterns in the Phallales species.

## 2. Materials and Methods

### 2.1. Sample Collection, DNA Isolation, and Sequencing

All Phallales samples newly sequenced in this study (*L. mokusin*, *P. indusiatus*, *P. hadriani*, *P. rigidiindusiatus*, and *P. rugulosus*) were collected from Guizhou, Yunnan, and Fujian, China (Table S1). The specimens were collected and preserved in sterile sealed bags at −20 °C prior to DNA extraction. Part of the samples were air-dried and then stored in sealed bags with silica gel desiccant in a climate-controlled drying cabinet to maintain long-term morphological and molecular integrity. The dried voucher specimens of strains *L. mokusin*, *P. indusiatus*, *P. hadriani*, *P. rigidiindusiatus*, and *P. rugulosus* were deposited in the laboratory of Guizhou Medical University under collection numbers FZ01, M398, M1052, MYII436, and M539, respectively. Morphological characteristics of these specimens were examined and documented. Genomic DNA was isolated using a Fungi Genomic DNA Extraction Kit (Solarbio, Beijing, China) and stored at −20 °C before sequencing. Whole-genome sequencing was performed via next-generation sequencing (Illumina HiSeq 4000 platform; 4 Gb raw data per specimen; Berry Genomic, Beijing, China).

For comparative analysis, the genome sequences of *P. dongsun* and *P. echinovolvatus* were retrieved from the NCBI database. The *L. mokusin* 2 and *P. indusiatus* 2 were obtained from the previously published studies: *L. mokusin* 2 was collected from Beijing [14], and *P. indusiatus* 2 was collected from Yibin, Sichuan Province [18]. By contrast, *L. mokusin* 1 and *P. indusiatus* 1 used in this study were newly collected from Guiyang, Guizhou Province, and Fuzhou, Fujian Province, respectively (Table S1), representing distinct geographical origins. Unless otherwise specified, *L. mokusin* and *P. indusiatus* in the text refer to the data generated in this study.

### 2.2. Assembly and Annotation of the Mitogenome

The mitochondrial genomes of five species were reconstructed using Geneious Prime v2023.2.1 [24]. Structural annotation was performed on the Galaxy platform (https://usegalaxy.org/, accessed on 10 March 2025) using MITOS [25] and Mfannot [26] (https://megasun.bch.umontreal.ca/apps/mfannot/, accessed on 10 March 2025) under the mold/protozoan mitochondrial genetic code Table S4. To ensure the accuracy of the annotation results, all predictions were manually verified. The 15 PCGs were predicted using the ORF Finder module in Geneious Prime v 2023.2.1 [24]. All annotated gene models were validated against published mitochondrial PCGs from homologous species. A physical map of the mitochondrial genome was generated with OGDraw v1.2 [27].

### 2.3. Sequence Analysis

The basic composition analysis of the mitogenome was performed with Geneious Prime v 2023.2.1 [24]. Strand asymmetry was assessed according to the formulas for AT skew [(A − T)/(A + T)] and GC skew [(G − C)/(G + C)]. MEGA 7.0 was employed to analyze codon usage [28]. The nonsynonymous (Ka) and synonymous (Ks) substitution rates for the PCGs of the Phallales species were calculated using DnaSP v6.12.03 [29]. Based on the Kimura 2-parameter (K2P) model, genetic distances among the 15 PCGs were estimated using MEGA 7.0 [28].

### 2.4. Phylogenetic Analysis

We downloaded mitochondrial genomes of 116 species spanning 10 orders of Basidiomycota from the NCBI database to reconstruct the phylogenetic tree. PCG and rRNA sequences were aligned separately using MAFFT v7.0 [30]. Poorly aligned homologous sites were then removed using trimAI [31] in PhyloSuite v1.2.2 [32] with default parameters. Subsequently, the resulting alignments were reviewed and manually refined using MEGA 7.0 [28]. The processed sequences were then assembled into three consolidated datasets with PhyloSuite v1.2.2 [32]. The first dataset (PCG12) consisted of the first and second codon positions from the 15 PCGs, totaling 8442 nucleotides. The second dataset (PCG12R) consisted of the PCG12 sequences and the two rRNA genes, comprising 10,929 nucleotides. The third dataset (AA) consisted of the amino acid sequences translated from the 15 PCGs, comprising 4221 amino acids. The optimal evolutionary models were selected via the PartitionFinder tool within PhyloSuite v1.2.2 under default parameters [32]. Specifically, the AA dataset was modeled under LG + I + G + F, whereas the GTR + I + G model was employed for the remaining datasets.

The phylogenetic trees were inferred through maximum likelihood (ML) and Bayesian inference (BI) analyses. Bayesian analysis was performed using MrBayes v3.2.7 [33] under default parameters, running four independent Markov chains for 1 million generations and sampling every 1000 generations. Convergence was confirmed when the average standard deviation of split frequencies fell below 0.01. The initial 25% of the sampled trees were discarded as burn-in, and the remaining trees were used to generate a majority-rule consensus tree and to estimate posterior probabilities. The ML analysis was conducted with IQ-TREE v1.6.12 [34], employing the ultrafast bootstrap approximation with 1000 replicates to assess branch support. Both the ML and BI trees were visualized in FigTree v1.4.4 https://tree.bio.ed.ac.uk/software/figtree/(https://github.com/rambaut/figtree/tags, accessed on 15 July 2025).

## 3. Results

### 3.1. Genome Features and PCGs of Phallales Mitogenomes

**Figure 1:**
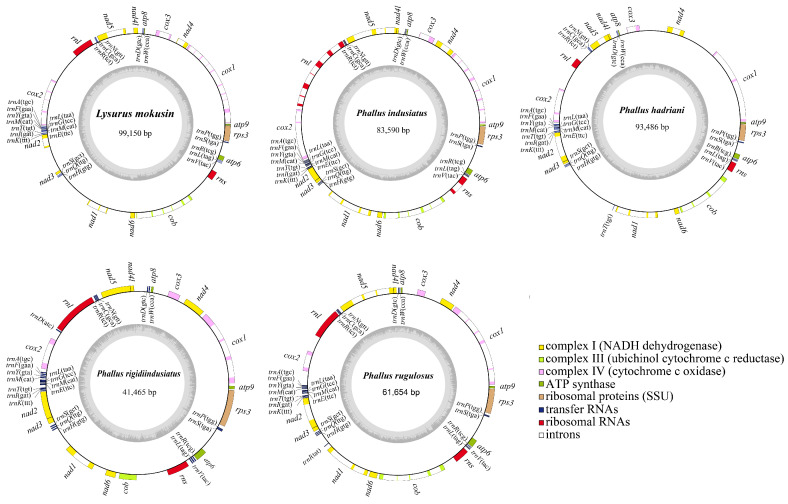
Circular maps of the mitogenomes of five Phallales mitogenomes. Genes are represented with different color blocks.

In the present study, we assembled five Phallales mitogenomes, all of which were circular DNA molecules. The size of *L. mokusin* was 99,150 bp, and those of *P. hadriani*, *P. indusiatus*, *P. rugulosus*, and *P. rigidiindusiatus* were 93,486 bp, 83,590 bp, 61,654 bp, and 41,465 bp, respectively (Figure 1). The length of the mitochondrial genome varies significantly across *Phallus*, with that of *P. hadriani* being 2.25 times the length of *P. rigidiindusiatus*. All five mitogenomes had a relatively low GC content. The *P. rugulosus* has the highest GC content at 25.1%. In contrast, the other four Phallales species sequenced have a lower GC content, ranging from 24.1% to 24.6% (Table 1). Both the AT and GC skew values of the five species were positive. Moreover, each mitogenome contained a complete set of PCGs (protein-coding genes), including *atp6*, *atp8*, *atp9*, *cob*, *cox1*-*3*, *nad1*-*6*, and *nad4L* for energy metabolism and *rps3* for transcriptional regulation.

Previous studies have reported the mitochondrial genomes of *L. mokusin* and *P. indusiatus* collected from different geographical regions. We conducted a comparative analysis of the mitochondrial genomes of *L. mokusin* and *P. indusiatus* from distinct geographic distributions (Figure S1). The mitochondrial genome of the *L. mokusin* strain examined in this study (designated *L. mokusin* 1) differs by 1985 bp from that of a previously reported Beijing strain (size 101,135 bp, designated *L. mokusin* 2) [14], with the variation concentrated primarily in the *rnl* gene and its adjacent region. Two fragments of 1793 bp and 315 bp are absent within the *rnl* gene of *L. mokusin* 1. Moreover, in the intergenic region between *rnl* and *cox2*, *L. mokusin* 1 possesses a tandem repeat structure composed of two 129 bp repeat units, whereas *L. mokusin* 2 contains only one such unit. Similarly, substantial mitochondrial genome structural variation was observed in *P. indusiatus* strains. The mitochondrial genome of the strain examined in this study (*P. indusiatus* 1) differs by 5549 bp from that of the Yibin-sourced strain (*P. indusiatus* 2) (*P. indusiatus* 1: 83,590 bp; *P. indusiatus* 2: 89,139 bp) [18]. This size difference is primarily due to the gain or loss of introns within the *rnl* and *cox1* genes. Specifically, *P. indusiatus* 1 lacks a 1153 bp intron in the *rnl* gene, and it lacks two introns (2921 bp and 1475 bp, respectively) within the *cox1* gene compared to *P. indusiatus 2*. These results indicated that the mitogenome size can vary considerably among strains from different geographical locations.

### 3.2. rRNA Genes and tRNA Genes in Phallales Mitogenomes

All five Phallales mitogenomes encoded two ribosomal RNA (rRNA) genes: the large subunit ribosomal RNA gene (*rnl*) and small subunit ribosomal RNA gene (*rns*) (Table 1). Among five Phallales fungi, the nucleotide lengths of the *rnl* gene varied considerably, ranging from 3163 bp to 3582 bp. A notable outlier was observed in *P. hadriani*, where the *rnl* gene was markedly truncated at 1403 bp. In contrast, the *rns* gene was highly conserved in length across most species, with sizes of 1518 bp, 1521 bp, 1516 bp, and 1519 bp in *P. hadriani*, *P. indusiatus*, *P. rugulosus*, and *P. rigidiindusiatus*, respectively, differing by only a few nucleotides. However, *L. mokusin* was an exception, with an *rns* gene length of 1481 bp, approximately 40 bp shorter than those of the other four species (Table S2). Furthermore, among the five Phallales fungi examined, only *P. indusiatus* contains one intron in its *rns* gene and six introns in its *rnl* gene, whereas the other four species possessed no introns in either *rnl* or *rns* genes. The presence of introns in ribosomal RNA genes also contributes to the higher intron abundance in *P. indusiatus* (Figure 1, Table 1).

The mitogenomes of *L. mokusin* and *P. indusiatus* each contain 24 tRNA genes, whereas those of *P. hadriani*, *P. rugulosus*, and *P. rigidiindusiatus* possess 25 tRNAs. Although tRNA gene lengths vary from 61 to 88 bp across all species, the majority fall within a narrow range of 71–74 bp, suggesting strong selective constraints to maintain structural and functional stability. Furthermore, among the 24 core tRNA genes, 20 are identical in length across all five species (*trnP*, *S*, *R*, *V*, *H*, *Q*, *S*, *K*, *I*, *T*, *E*, *M*, *G*, *L*, *Y*, *F*, *A*, *R*, *N*, *D*). Three tRNA genes show minor size variations of 1–2 bp (*trnM*, *C*, *W*) (Table S2). The conservation of tRNA gene lengths may be associated with the maintenance of their structural and functional integrity. A notable exception is *trnL*, which is drastically reduced to 61 bp in *L. mokusin* and *P. rugulosus* (Table S2). In contrast, this gene is 85 bp in length in the three other species examined here, as well as in all other publicly available Phallales mitogenomes (*P. echinovolvatus* and *P. dongsun*), suggesting a specific structural evolutionary event within this lineage.

In the five Phallales mitogenomes, tRNA genes formed six clusters localized in distinct intergenic regions between *atp8* and *nad4L*(*trnW*, *D*), *nad5* and *rnl* (*trnR*, *C*, *N*), *cox2* and *nad2* (*trnA*, *F*, *Y*, *M*, *L*, *G*, *M*, *E*, *T*, *I*, *K*), *nad3* and *nad1* (*trnS*, *Q*, *H*), *rns* and *atp6* (*trnV*, *L*, *R*), and *atp6* and *rps3* (*trnS*, *P*) (Figure 1). These tRNA genes collectively encode all 20 standard amino acids, each folding into the classical cloverleaf secondary structure. Notably, within the five Phallales mitogenomes, leucine, arginine, and serine are each encoded by two distinct tRNA genes, with each pair harboring unique anticodons. By contrast, two methionine-encoding tRNA genes share identical anticodons (Figure 1; Table S2). Interestingly, *P. hadriani* also possesses two distinct threonine-tRNA genes (*trnT*) with identical anticodons, *P. rugulosus* harbors two isoleucine-tRNA genes (*trnI*) each with a unique anticodon, and *P. rigidiindusiatus* contains two aspartate-tRNA (*trnD*) genes each with a distinct anticodon (Figure 1; Table S2). This explains the presence of 25 tRNA genes in their respective mitogenomes.

### 3.3. Analysis of Codon Usage

**Figure 2:**
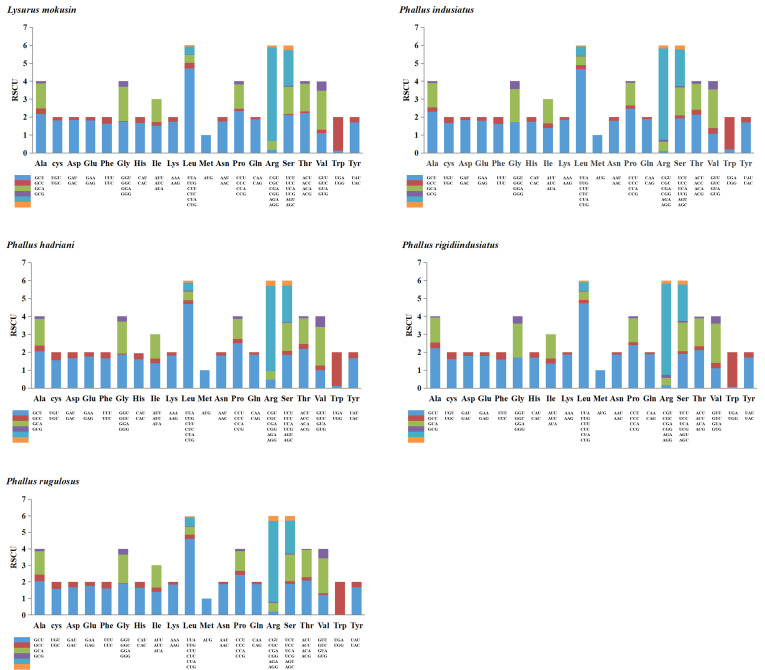
Codon usage analysis of 15 protein-coding genes in five Phallales mitogenomes. Axes: codon families (below *x*-axis) and their usage frequency (*y*-axis).

For the five Phallales species in this study, the most frequently used start codon among core protein-coding genes is ATG, followed by TTA and ATT (Table S3). Start codon usage bias varies among different fungal species. For example, the most frequently used start codon in the core protein-coding genes of *L. mokusin* is ATT, whereas those of *P. rugulosus* and *P. hadriani* prefer TTA. In *P. rigidiindusiatus*, all core protein-coding genes initiate with ATG. TAA is the most frequently used stop codon among the five Phallales species. In contrast, the *cob* and *cox2* genes of *P. hadriani* and the *nad2* gene of *L. mokusin* are exceptions in employing TAG as the stop codon (Table S3). Codon usage analysis (Figure 2; Table S4) indicated that the most frequently used codons in all five Phallales mitogenomes were TTA (for leucine; Leu), followed by TTT (for Phenylalanine; Phe), AAT (for asparagine; Asn), ATT (for isoleucine; Ile), and ATA (for isoleucine; Ile). This suggests that protein-coding genes among Phallales species exhibit highly conserved patterns of amino acid usage. Interestingly, in *P. indusiatus*, the codons AAT and ATT exhibit the same usage frequency (Table S4), which may reflect certain specificities in the evolutionary demand for these amino acids. The highly frequent use of A and T in codons contributed to a relatively high AT content (>70%) in the five Phallales mitogenomes.

### 3.4. Mitogenome Composition Analysis

**Figure 3:**
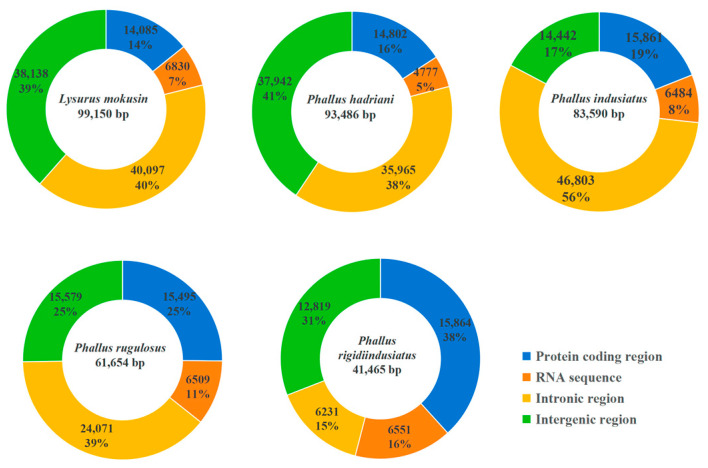
The protein-coding region, RNA coding region, intronic region, and intergenic region in the five Phallales species.

In *P. hadriani*, intergenic regions constituted the largest proportion (41%) of the mitogenome, followed closely by intronic regions (38%) (Figure 3). In *L. mokusin*, *P. indusiatus*, and *P. rugulosus*, intronic regions comprised the largest component of the mitogenomes, totaling 40%, 56%, and 39%, respectively. Interestingly, intronic regions represented the smallest share of the mitogenome (15%) in *P. rigidiindusiatus*, and the protein-coding region was the largest component (38%). We also observed a negative correlation between the mitogenome size and the proportional content of protein-coding genes. A similar trend was observed for the size of RNA-coding regions. This permits highly efficient utilization of the genome for encoding proteins and RNAs. These results indicated that the intronic region and intergenic region were important factors related to the mitogenome expansion of the five Phallales species.

### 3.5. Analysis of Intron Insertion Sites

Introns are present in the mitogenomes of all five Phallales species, with intron counts varying significantly among them. The *L. mokusin* contains the highest number of introns (24) in the 15 PCGs, while *P. rigidiindusiatus* has the fewest (5). Notably, the *atp6*, *atp8*, *atp9*, *nad3*, *nad4L*, *nad6*, and *rps3* genes lack introns in all examined species. The *cox1* gene harbors relatively more intron insertion sites, with *L. mokusin* showing the maximum (9). Overall, intron insertions are less frequent in *nad2*, *nad4*, and *cox3*, with no more than one intron present in these three genes across the five species (Table S5).

### 3.6. Variation and Evolutionary Rates of Core PCGs

**Figure 4:**
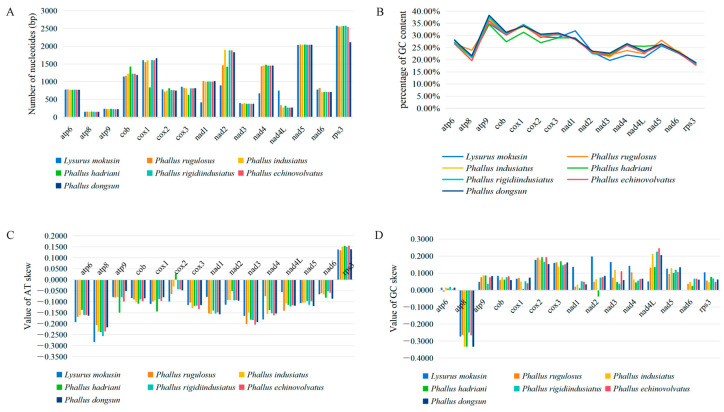
Comparison of gene length and base composition of the 15 PCGs among seven Phallales mitogenomes. (**A**) Variation in gene length; (**B**) GC content; (**C**) AT skew; (**D**) GC skew.

To provide a more comprehensive understanding of the nucleotide variability and evolutionary rates of core PCGs in the mitochondrial genomes of the Phallales species, a total of seven Phallales species were analyzed in this study. Among them, five species were newly sequenced in the present study, and the sequences of the other two species (*P. dongsun* and *P. echinovolvatus*) were obtained from the GenBank database. The 15 PCGs examined across the seven Phallales species displayed variations in sequence length (Figure 4). However, *atp6*, *atp8*, and *nad3* exhibited length conservation, with identical sizes observed in five of the seven Phallales species. In contrast, the *nad2* gene showed the largest length variation, up to 996 bp. Notably, 14 PCGs exhibited identical lengths between *P. rigidiindusiatus* and *P. echinovolvatus*, with only the *rps3* gene differing by 30 bp. Regarding GC content, *atp9* had the highest average value at 36.13%, followed by *cox1* with 33.67%, whereas *rps3* had the lowest average GC content at 18.24%. Among the 15 PCGs, only *rps3* showed a positive AT skew across the seven Phallales species, suggesting a general trend toward T-richness rather than A-richness on the leading strand of PCGs. Except for *atp8*, which has a negative GC skew, all PCGs had a positive GC skew (Figure 4).

**Figure 5:**
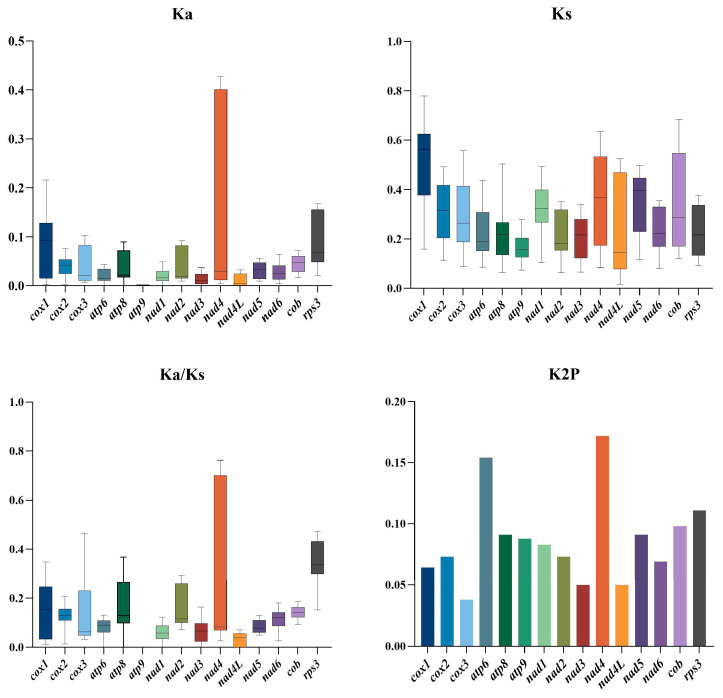
Genetic variation in the 15 PCGs across seven Phallales species. Ka, rate of nonsynonymous substitutions per nonsynonymous site; Ks, rate of synonymous substitutions per synonymous site; Ka/Ks, the ratio of Ka to Ks; K2P, genetic distance calculated under the Kimura 2-parameter model.

We compared the non-synonymous substitution rate (Ka) of the 15 conserved PCGs in the seven Phallales mitogenomes (Figure 5). The *cox1* gene had the highest Ka value, followed by *rps3*, whereas *atp9* demonstrated the lowest Ka value (Ka = 0). The *cox1* gene also exhibited the highest synonymous substitution (Ks) rate, while *nad4L* had the lowest Ks rate. Among the 15 PCGs, *atp9* displayed the lowest Ka/Ks ratio, while *rps3* showed the highest Ka/Ks ratio. These results suggest that the *atp9* gene had undergone stronger evolutionary selection pressure, while the *rps3* gene experienced the lowest selective constraint. All 15 PCGs exhibited Ka/Ks ratios below 1, providing strong evidence that purifying selection has been the predominant evolutionary force acting on these genes across the Phallales species. The average Kimura 2-parameter (K2P) genetic distance analysis revealed that *nad4* has the largest value, followed by *atp6* and *rps3*. The results indicated that these genes had diverged significantly during evolution. However, *cox3* had the smallest K2P value, followed by *nad4L*, suggesting these genes are highly conserved.

### 3.7. Gene Order Analysis of Phallales

**Figure 6:**
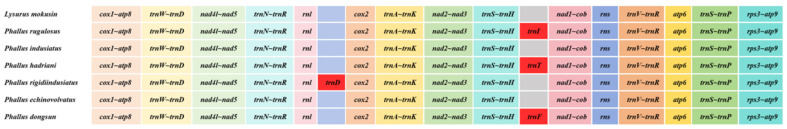
Mitochondrial gene arrangement analyses of the seven Phallales species. The *cox1*-*atp8* represents *cox1*-*nad4*-*cox3*-*atp8*; the *trnN*-*trnR* represents *trnN*-*trnC*-*trnR*; the *trnA*-*trnK* represents *trnA*-*trnF*-*trnY-trnM*-*trnL*-*trnG*-*trnM-trnE*-*trnT*-*trnI*-*trnK;* the *trnS*-*trnH* represents *trnS*-*trnQ*-*trnH*; the *nad1*-*cob* represents *nad1*-*nad6*-*cob*; and the *trnV*-*trnR* represents *trnV*-*trnL*-*trnR.* Conversed gene order are shown in same color blocks. Red blocks are used to highlight different gene doubling events.

Gene order analysis was performed on seven Phallales species to investigate mitogenomic rearrangement patterns. Among them, five species were newly sequenced in this study, and the other two species were derived from previously published data, allowing for a broader and more reliable comparison of gene order evolution within the Phallales. The results revealed that all the examined Phallales species have identical gene order of PCGs and rRNA genes, consistently arranged as *cox1*-*nad4*-*cox3*-*atp8*-*nad4L*-*nad5*-*rnl*-*cox2*-*nad2*-*nad3*-*nad1* -*nad6*-*cob*-*rns*-*atp6*-*rps3*-*atp9* (Figure 6). As far as tRNA genes are concerned, four tRNA doubling events have occurred in all Phallales mitogenome, including *trnM*, *trnL*, *trnR*, and *trnS*. This may represent an adaptive response to distinctive environmental selective pressures during the evolution or correlate with its derived biological traits. Within the mitochondrial genomes of all seven examined species in Phallales, 24 tRNA genes are arranged in identical order. Moreover, the mitogenome of *P. rugulosus*, *P. hadriani*, *P. rigidiindusiatus*, and *P. dongsun* each had another tRNA gene doubling event involving *trnI*, *trnT*, *trnD*, and *trnF* gene, respectively. The preserved gene arrangement in mitochondrial genomes across the Phallales species demonstrates evolutionarily persistent architectural stability within this order.

### 3.8. Phylogenetic Relationship Analysis

**Figure 7:**
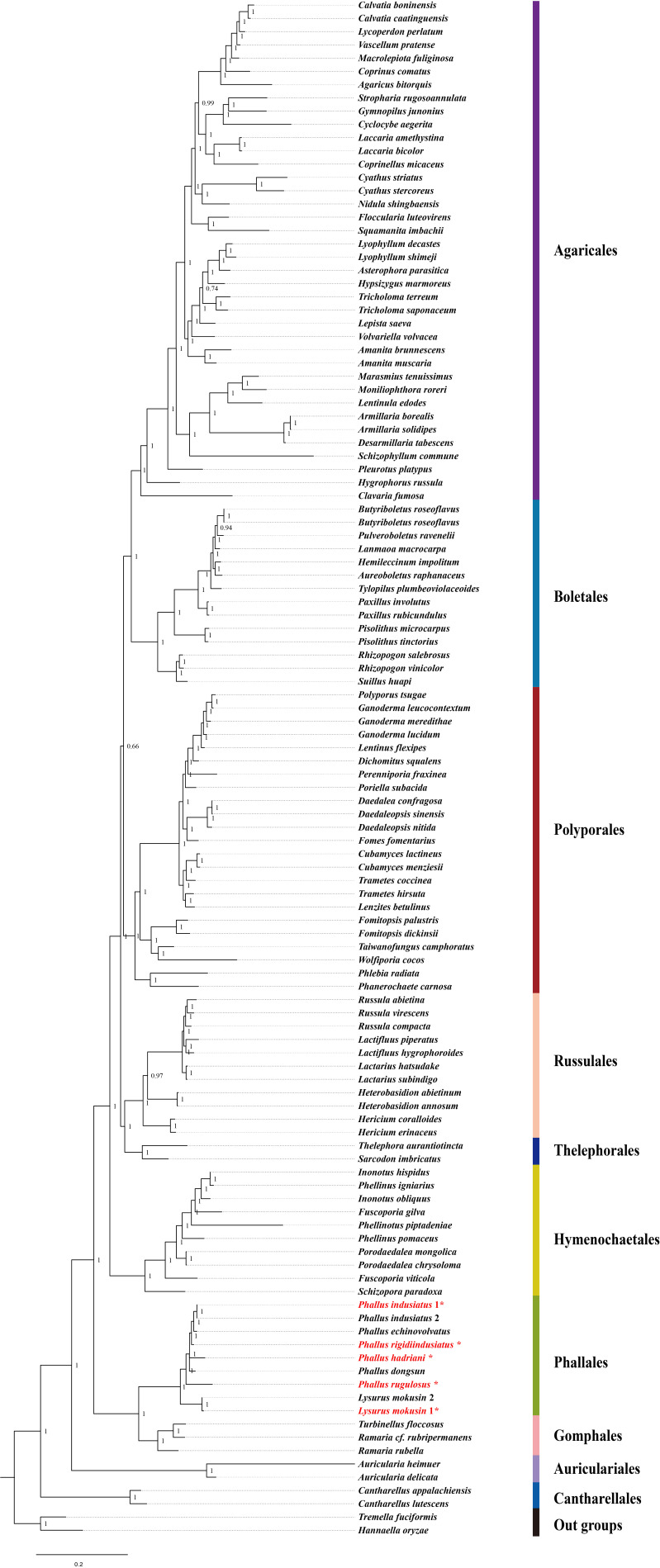
Phylogenetic analysis of 114 Agaricomycetes species using MrBayes based on 15 PCGs and 2 rRNA sequences. Numbers at the nodes represent posterior probability values from Bayesian inference. Species marked with an asterisk (*) represent data generated in this study. Strains labeled 1 and 2 originate from different geographic origins, as detailed in Methods. For the species and NCBI accession numbers employed in the phylogenetic analyses, refer to Supplementary Table S6.

We investigated the phylogenetic relationships of 114 Agaricomycetes species based on the concatenated mitochondrial gene sequences. The phylogenetic tree topologies inferred from different datasets and inference methods were compared to assess the robustness of the results. All six phylogenetic trees (ML and BI based on PCG12, PCG12R, and AA datasets) displayed highly consistent topological structures, indicating the robustness of our phylogenetic inference (Figure 7 and Figures S2–S6). The PCG12R dataset, which combines protein-coding genes and rRNA genes, provides more comprehensive phylogenetic information for clarifying relationships among closely related taxa. Notably, the Bayesian tree inferred from the PCG12R dataset provided the highest phylogenetic resolution and robustly resolved the relationship between *P. dongsun* and *P. hadriani* with maximum support (BPP = 1.00). Therefore, the PCG12R Bayesian tree was selected as the main phylogenetic tree for display in this study. *Tremella fuciformis* and *Hannaella oryzae* were selected as out-groups. The 114 Agaricomycetes species (Table S6) were clustered into 10 groups, corresponding to the orders of Cantharellales, Auriculariales, Agaricales, Boletales, Polyporales, Hymenochaetales, Russulales, Thelephorales, Gomphales, and Phallales. The phylogenetic analysis strongly supported that Agaricales and Boletales are sister taxa (BPP = 1.0, BS = 100%) (Figure 7 and Figures S2–S6). The orders of Phallales and Gomphales were clustered on the same branch with a support value of 1, indicating that these two orders are closely related. These results are consistent with previous studies, supporting the reliability of the phylogenetic tree [18].

Within the Phallales clade, *L. mokusin* first clustered into a subclade with a support value of 1.0, and then clustered with species of the genus *Phallus*. The genera *Phallus* and *Lysurus* fromed distinct branches, indicating significant divergence in mitochondrial genome evolution among major lineages of the Phallales. Within the *Phallus* genus, *P. indusiatus* first clustered into a subclade, and then formed a well-supported cluster with the other five Phallus species (*P. dongsun*, *P. hadriani*, *P. rigidiindusiatus*, *P. echinovolvatus*, and *P. rugulosus*). The consistent branch support value of 1 implies a close evolutionary affinity among these *Phallus* species. Moreover, *P. rugulosus* formed a distinct clade, while the other five species (*P. dongsun*, *P. hadriani*, *P. rigidiindusiatus*, *P. echinovolvatus*, and *P. indusiatus*) clustered together in a separate clade. This result agrees with previous phylogenetic analyses based on *ITS*–*LSU* sequences [13], suggesting that the mitochondrial genome was an effective molecular marker for inferring evolutionary relationships in Agaricomycetes.

## 4. Discussion

Mitochondria are widely present in eukaryotic cells and involved in key life processes such as energy conversion, cellular signaling pathways, and amino acid metabolism, as well as the regulation of cellular senescence and death [35,36]. Due to its advantages, including a relatively fast evolutionary rate, relatively stable composition, small length, and simple structure, the mitochondrial genome has become one of the most widely used and informative genetic markers in population and evolutionary research [15,16,37]. It is worth noting that although fungi represent a group with extremely high diversity, research on their mitochondrial genomes remains insufficient. In this study, five mitochondrial genomes of the Phallales species, including *L. mokusin*, *P. indusiatus*, *P. hadriani*, *P. rigidiindusiatus*, and *P. rugulosus*, were sequenced, assembled, and analyzed. Notably, the characteristics of the mitochondrial genomes of *P. hadriani*, *P. rigidiindusiatus*, and *P. rugulosus* are reported here for the first time.

Fungal mitogenomes exhibit significant size variation, with a range from 11 kb (*Hanseniaspora uvarum*) [38] to 332.1 kb (*Golovinomyces cichoracearum*) [39]. Previous studies have indicated that the mitochondrial genome sizes of other species in the Phallales are 50,098 bp (*P. echinovolvatus*) [18] and 59,243 bp (*P. dongsun*). Our study revealed that the mitogenome size in the Phallales fungi varies widely, from 41,465 to 99,150 bp. Strikingly, the mitogenome size of *L. mokusin* is 2.39 times larger than that of *P. rigidiindusiatus*. Moreover, variations in introns, intergenic regions, repetitive sequences, and the accumulation of horizontally transferred genes are key factors responsible for the differences in the mitochondrial genome size [21,22,40,41]. Among these, introns were identified as the most important factor in the variation in mitogenome size in *Phallus* [18]. Our analysis of five Phallales species revealed distinct compositional patterns in their mitogenomes. *P. hadriani* displayed the highest proportion of intergenic regions, accounting for 41% of its mitogenomes. In contrast, intronic regions constituted the majority of the mitogenomes in *L. mokusin* (40%), *P. indusiatus* (56%) and *P. rugulosus* (39%). Notably, *P. rigidiindusiatus*, possessing the smallest mitogenome, exhibited the highest relative proportion of protein-coding regions (38%). Furthermore, we observed a positive correlation between the mitogenome size and the cumulative length of intronic and intergenic sequences across the Phallales order. In conclusion, introns and intergenic regions serve as the main factor that drives variations in mitogenome size among the Phallales species.

Our study further revealed that mitogenome size variation exists even among strains of the same species derived from different geographic regions. In *L. mokusin*, the *rnl* gene and a 129 bp tandem repeat sequence located in the intergenic region substantially contributed to the observed variation in mitochondrial genome size. Whether the observed mitogenomic variation influences the morphology of *L. mokusin*, such as the number of ridges (typically 4–6) on its stipe, remains unknown and requires further investigation with expanded sampling. In *P. indusiatus*, the size variation in mitochondrial genomes among strains was primarily attributed to the loss or gain of introns within the *rnl* and *cox1* genes. This finding aligns with previous reports. For instance, frequent intron loss/gain events have also been identified as a major factor underlying mitogenome divergence among strains in *Cordyceps militaris*, *Ganoderma lingzhi*, and *Cyathus Striatus* [42,43,44]. These cases demonstrate that mitogenomes can undergo dynamic changes even within a single species. Further supporting this, a study of eight *Diaporthe longicolla* isolates from different U.S. states revealed a maximum mitogenome size difference of up to 5746 bp, with variations in intron number and repetitive sequences also observed among geographical regions [45]. Collectively, this evidence leads us to hypothesize that intraspecific variation in mitochondrial genomes may be a common phenomenon, potentially associated with factors such as geographical isolation and ecological adaptation. To test this hypothesis, we will collect specimens from a wider range of geographic areas for further analysis.

In this study, the mitochondrial ribosomal RNA genes of seven species from the order of Phallales were analyzed (Table S2). The *rnl* gene exhibited significant size divergence across the seven Phallales species (*L. mokusin*, *P. hadriani*, *P. indusiatus*, *P. rugulosus*, *P. rigidiindusiatus*, *P. echinovolvatus*, and *P. dongsun*), with lengths ranging from 3163 bp to 3582 bp. Notably, the gene in *P. hadriani* was drastically reduced to 1403 bp, which is substantially shorter than the minimum size range found in its congeners (3163 bp). This pronounced contraction may suggests a specific evolutionary event, such as a major deletion or gene rearrangement. In contrast, the *rns* gene displayed strong evolutionary conservation, varying by only 3–5 bp among *P. hadriani*, *P. indusiatus*, *P. rugulosus*, *P. rigidiindusiatus*, *P. echinovolvatus*, and *P. dongsun* (1516–1521 bp). The considerably shorter *rns* gene in *L. mokusin* (1481 bp), approximately 40 bp smaller than that of its congeners, may reflect lineage-specific evolutionary dynamics, such as accelerated structural evolution or distinct selective constraints.

The content of the core genes in fungal mitochondrial genomes is generally conserved, whereas the gene order exhibits remarkable plasticity [16,21,22]. We compared the mitochondrial gene order of all seven sequenced Phallales species. The stability of PCGs and rRNA gene arrangement in Phallales mitogenomes reflects their essential role in mitochondrial function. However, tRNA (*trn*) genes, which encode amino acids, are considered conserved in the mitochondrial genomes of fungi within the same order [16,46]. We analyzed the species of Phallales with known mitogenomic sequences and found that *trn* genes in mitochondrial genomes exhibited extremely high length conservation, with only 1–2 bp variations, except the *trnL* gene of *L. mokusin* and *P. rugulosus*. The phylogenetic tree showed that *L. mokusin* and *P. rugulosus* occupied a basal, early-branching clade, marking them as early-diverging lineages. This topological placement indicates that the longer *trnL* (85 bp) represents a derived trait that emerged during subsequent evolution, and this variation was likely an adaptive change favored by natural selection. The duplication of *trn* genes was observed in many mitochondrial genomes of fungi and is thought to serve as an adaptive strategy, potentially improving the process of mitochondrial translation and supporting increased cellular energy production [22,23]. Our study revealed that the duplication of *trnL*, *trnM*, *trnR*, and *trnS* is present in the mitochondrial genomes of all seven sequenced Phallales species. This finding indicates that these *trn* duplications represent a synapomorphy evolved during the evolutionary history of Phallales, which may play an irreplaceable role in maintaining mitochondrial function or facilitating adaptive evolution of this order. Notably, the gene order of all PCGs, ribosomal RNA genes, and 24 *trn* genes is completely identical across the mitochondrial genomes of Phallales species, with the only exception being the presence of additional *trnI*, *trnT*, *trnD*, and *trnF* duplications in four species, namely *P. rugulosus*, *P. hadriani*, *P. rigidiindusiatus* and *P. dongsun*. This evolutionary conservation indicates that strong purifying selection is acting to constrain structural changes.

Intronic sequences are widespread in fungal mitochondrial genomes. Previous studies have revealed that fungal mitochondrial genomes contain a substantial number of group I introns, notably within the *cox1*, *cob*, and *rnl* genes [47,48]. Introns are considered to be mobile genetic elements involved in genomic gain and loss events [49,50,51]. They can spread to intron-free target sites via homing, but they are also susceptible to loss through sporadic deletion or gene conversion events [50,51]. Our study revealed considerable variation in intron abundance and localization within the mitochondrial genomes of species belonging to Phallales. Notably, the *cox1* and *cob* genes were found to be particularly intron-rich, indicating frequent gain and loss events during their evolution. Furthermore, among all sequenced species within the Phallales, the *rns* and *rnl* genes of *P. indusiatus* are uniquely characterized by the presence of introns, which are absent in all other representatives of this order. Phylogenetic analysis reveals that *P. indusiatus* occupies a derived position within the evolutionary tree. These findings collectively suggest that the introns in the ribosomal RNA genes of the *P. indusiatus* mitochondrial genome were likely recently acquired, potentially via horizontal transfer.

Species of the order Phallales have a global distribution and exhibit a spectrum from edible to toxic. Their morphological conservation makes accurate identification difficult. Traditionally, phylogenetic studies of fungi have relied on nuclear markers, such as the *ITS*, *LSU*, and *RPB2* genes. With the advancement of mitogenomic research, mitochondrial genes have been widely employed as valuable markers, providing useful information for inferring phylogenetic relationships among fungal species [16]. For example, studies have employed the *atp6*, *LSU*, and *RPB2* genes from the *Lysurus* and *Phallus* species to build a phylogenetic tree [14]. Mitochondrial genes have also been employed as a combined dataset constructed from all conserved genes for phylogenetic analysis [21,52,53]. The phylogenies of major fungal lineages based on mitochondrial data are similar to those inferred from nuclear datasets [53]. In this study, we constructed a phylogenetic tree based on a set of 15 conserved PCGs and rRNA genes in Phallales and nine other Agaricomycetes orders. The species of the Phallales order share a closer phylogenetic relationship with the species of the Gomphales order. Moreover, the phylogenetic analysis revealed that *P. rugulosus* forms a distinct, well-supported clade within the *Phallus* genus, clearly separated from the other five congeners. This result is congruent with the topology produced from the nuclear *LSU*–*ITS* dataset [13]. This clear genetic divergence suggests that *P. rugulosus* may represent a unique evolutionary lineage. Discordances were also observed between the mitochondrial genome-based tree in our study and established nuclear phylogenies [13]. Therefore, an integrated taxonomic framework, incorporating morphological characteristics, ecological data, and additional molecular markers, is crucial for reliably delineating species in the Phallales order.

## 5. Conclusions

This study successfully sequenced and characterized five mitochondrial genomes of the Phallales order, three of which are newly reported. These mitochondrial genomes exhibited substantial variations in genetic content, gene lengths, tRNA composition, and codon usage. Introns and intergenic regions are significant factors influencing the size of mitochondrial genomes in these fungi. Our comparative mitochondrial genomic analysis revealed that the gene order is highly conserved in seven sequenced Phallales fungi, encompassing 15 core PCGs, 2 rRNA genes, and 24 tRNA genes. An additional tRNA duplication events were identified in *P. rugulosus*, *P. hadriani*, *P. rigidiindusiatus*, and *P. dongsun*. The phylogenetic analysis showed that species from Phallales and Gomphales share a closer evolutionary relationship. However, for the genus *Phallus*, the phylogenies inferred from mitochondrial and nuclear gene sequences are not fully congruent. The resolution of this discordance is therefore critical for elucidating the phylogenetic relationships within the genus. Our findings establish a foundational framework for future studies on the evolution, genetics, and taxonomy of Phallales and related fungal orders.

## Figures and Tables

**Table 1:** Comparison of five species of Phallales mitogenomes.

Genome Features	*Lysurus mokusin*	*Phallus hadriani*	*Phallus rugulosus*	*Phallus indusiatus*	*Phallus rigidiindusiatus*
Total size (bp)	99,150	93,486	61,654	83,590	41,465
Overall GC (%)	24.6	24.5	25.1	24.5	24.1
GC-skew	0.0929	0.1	0.0955	0.0891	0.094
AT-skew	0.0215	0.0225	0.0171	0.0317	0.0002
No. PCGs	15	15	15	15	15
No. tRNA	24	25	25	24	25
No. rRNA	2	2	2	2	2
No. introns	24	14	16	30	5
Exonic regions (bp)	14,085	14,802	15,495	15,861	15,864
Intergenic regions (bp)	38,138	37,942	15,579	14,442	12,819
Intronic regions (bp)	40,097	35,965	24,071	46,803	6231

## Data Availability

The complete mitogenomes of *Lysurus mokusin*, *Phallus indusiatus*, *Phallus hadriani*, *Phallus rigidiindusiatus*, and *Phallus rugulosus* were deposited in NCBI GenBank following the accession numbers in the Supplementary Materials.

## Supplementary Materials

The following supporting information can be downloaded at: https://www.mdpi.com/article/10.3390/jof12030207/s1, Figure S1: Schematic representation of the major structural differences in the mitochondrial genomes among *L. mokusin* and *P. indusiatus* strains. Figure S2: The phylogenetic tree of AA dataset constructed using MrBayes; Figure S3: The phylogenetic tree of PCG12 dataset constructed using MrBayes; Figure S4: The phylogenetic tree of AA dataset constructed using IQ-TREE; Figure S5: The phylogenetic tree of PCG12 dataset constructed using IQ-TREE; Figure S6: The phylogenetic tree of PCG12R dataset constructed using IQ-TREE; Table S1: Collecting information; Table S2: Comparison of rRNA genes and tRNA genes in Phallales mitogenomes; Table S3: Start and stop codons analysis of five species from Phallales; Table S4: Codons usage analysis of five species from Phallales; Table S5: Analysis of intron insertion sites within core PCGs in five Phallales species; Table S6: Species used for phylogenetic analysis in this study.

## Abbreviations

The following abbreviations are used in this manuscript:

mitogenome	mitochondrial genome
*ITS*	internal transcribed spacer
*LSU*	large subunit ribosomal DNA
*RPB2*	RNA polymerase II subunit 2
PCG	protein-coding gene
tRNA	transfer RNA
rRNA	ribosomal RNA
*nad1*-*6*	NADH dehydrogenase 1-6
*nad4L*	NADH dehydrogenase 4L
*cox1*-*3*	cytochrome c oxidase 1-3
*cob*	cytochrome b
*atp6*	ATP synthase 6
*atp8*	ATP synthase 8
*atp9*	ATP synthase 9
*rnl*	large subunit ribosomal RNA
*rns*	small subunit ribosomal RNA
*rps3*	ribosomal protein gene
Ka	nonsynonymous substitution rate
Ks	synonymous substitution rate
K2P	Kimura-2-parameter
ML	maximum likelihood
BI	Bayesian inference
bp	base pair
kb	kilo base pair
Gb	giga base pair
GC	guanine-cytosine
AT	adenine-thymine
BPP	Bayesian Posterior Probability
BS	bootstrap support
