# Supplementary material for: Phylogenetic Relationships of Five Phallales Species Based on Mitochondrial Genome Analysis
Source: J Fungi (Basel). 2026 Mar 13;12(3):207. doi: 10.3390/jof12030207 (PMC13027951; doi:10.3390/jof12030207)
Supplement: Supplementary file 1 [file jof-12-00207-s001.zip › supplementary files.pdf]

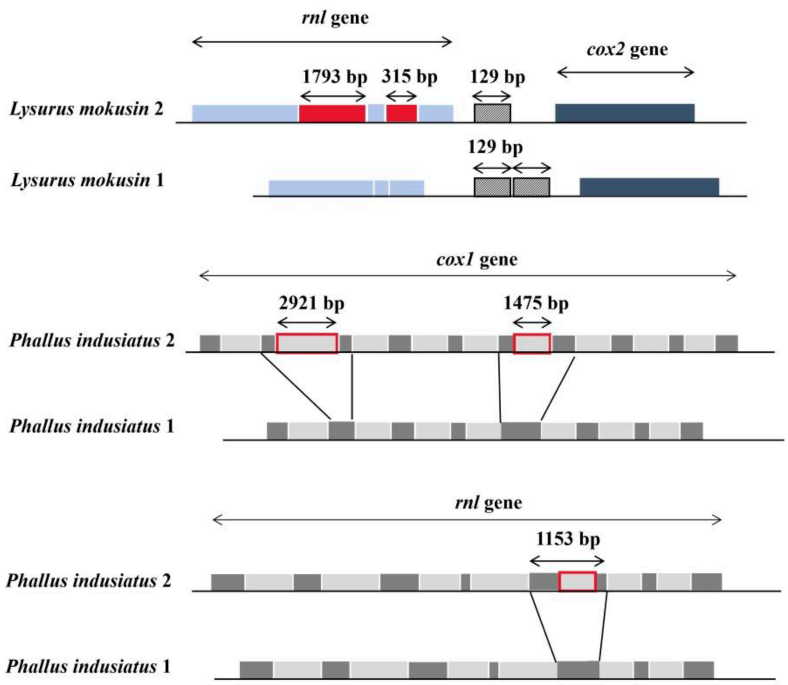

Figure S1. Schematic representation of the major structural differences in the mitochondrial genomes among *L. mokusin* and *P. indusiatus* strains. For *L. mokusin*, red blocks denote sequence loss/gain, and shaded areas represent repetitive regions. For *P. indusiatus*, exons and introns are shown in dark and light grey, respectively, with intron loss/gain specifically marked by red boxes. Strains labeled 1 and 2 originate from different geographic origins, as detailed in the Methods.

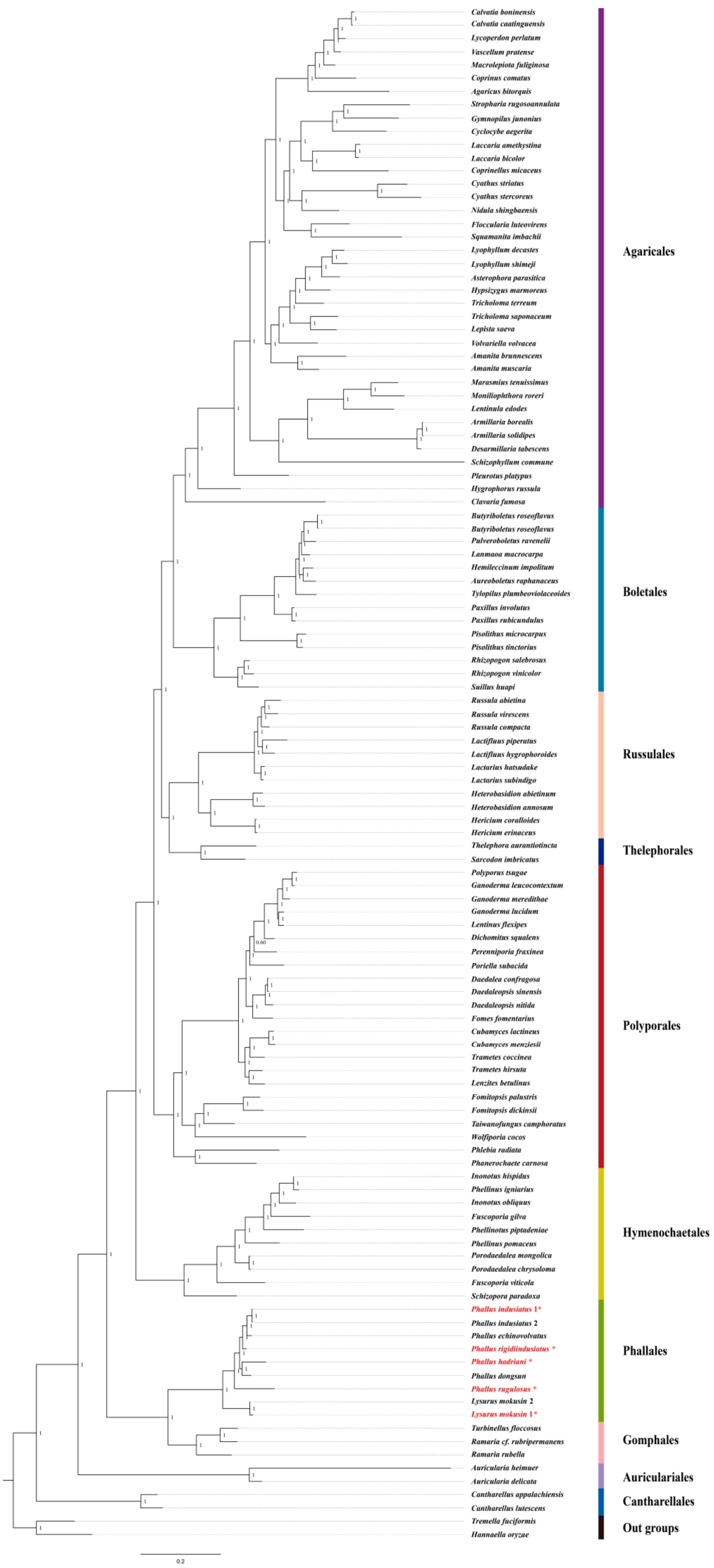

Figure S2. The phylogenetic tree of AA dataset constructed using MrBayes.

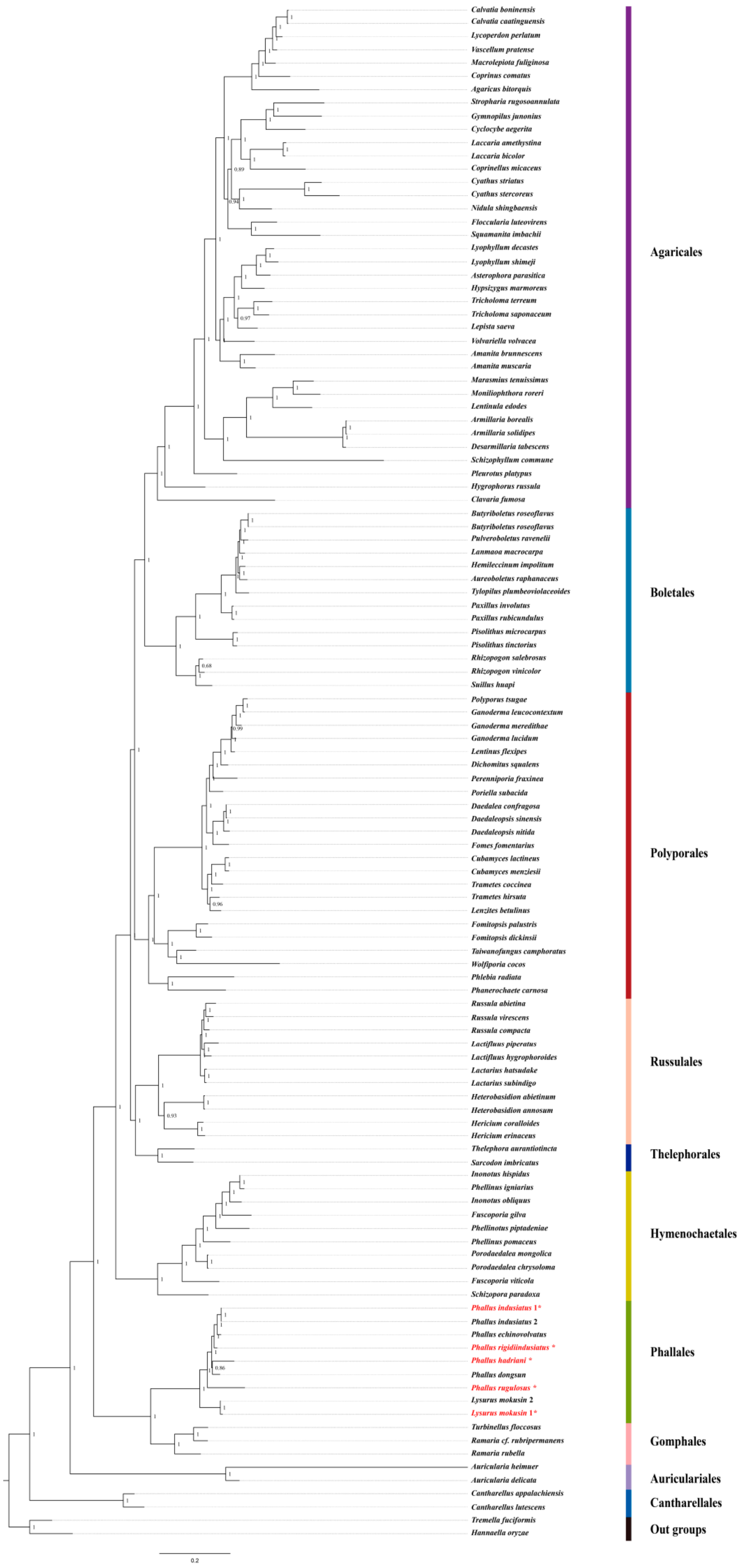

Figure S3. The phylogenetic tree of PCG12 dataset constructed using MrBayes.

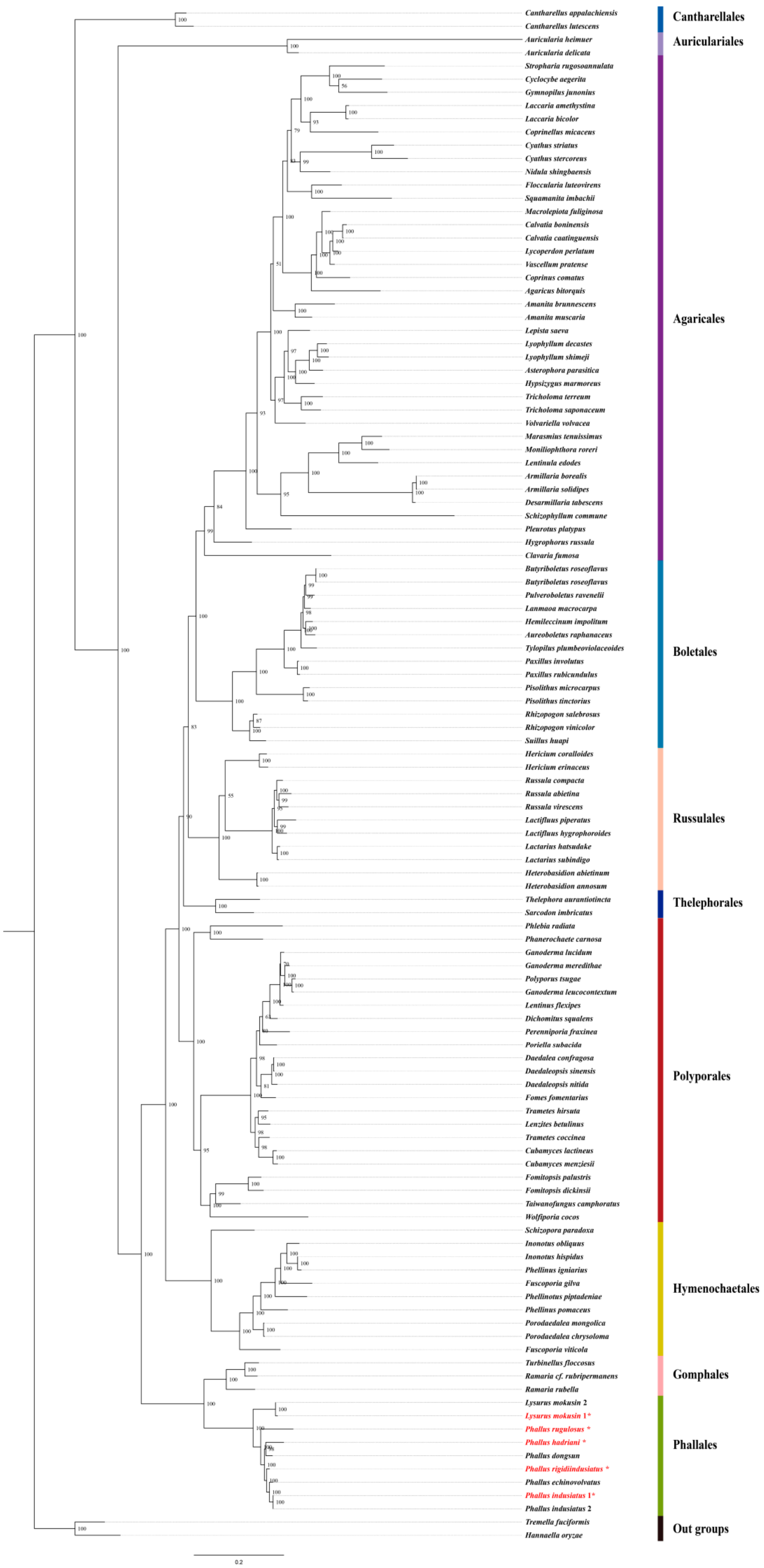

Figure S4. The phylogenetic tree of AA dataset constructed using IQ-TREE.

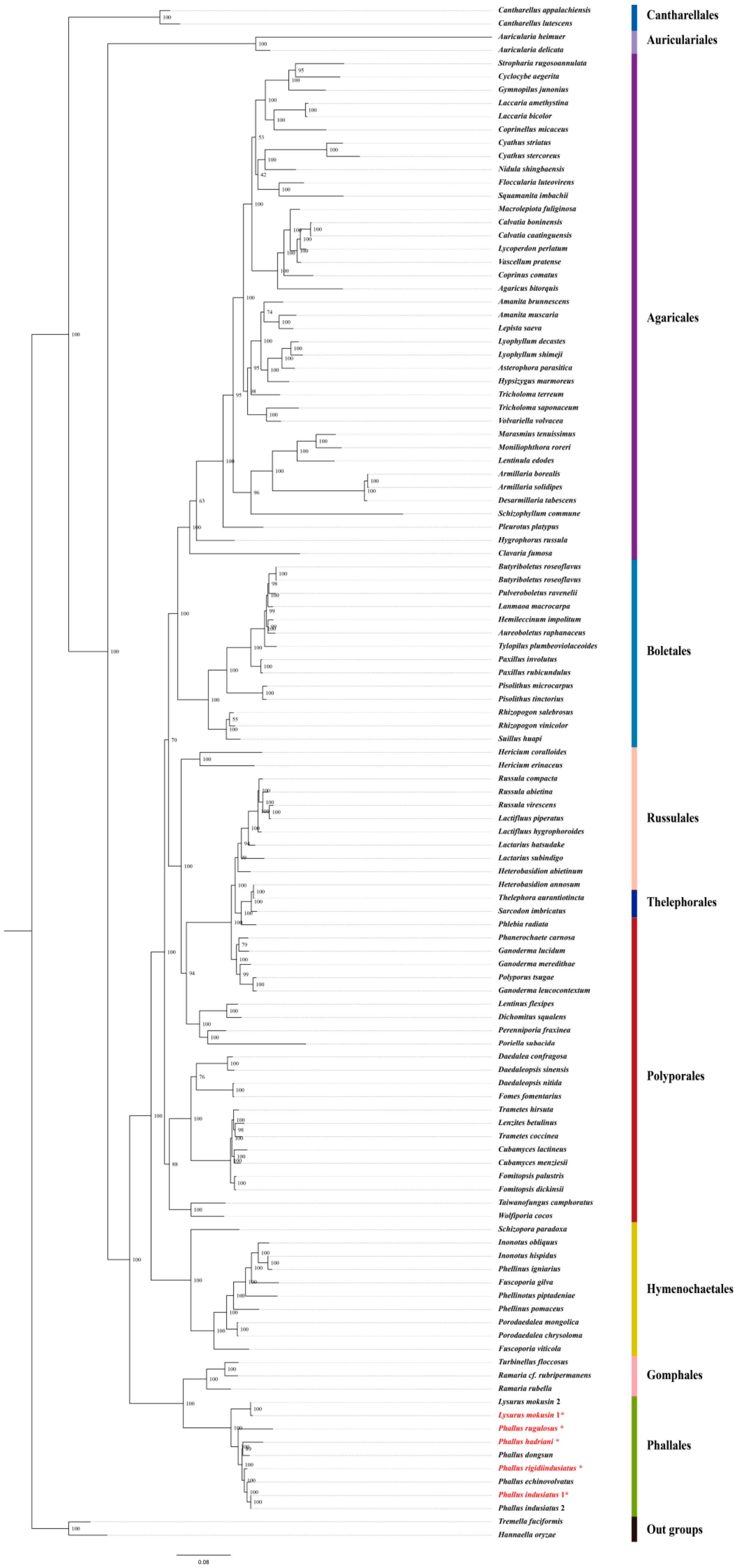

Figure S5. The phylogenetic tree of PCG12 dataset constructed using IQ-TREE.

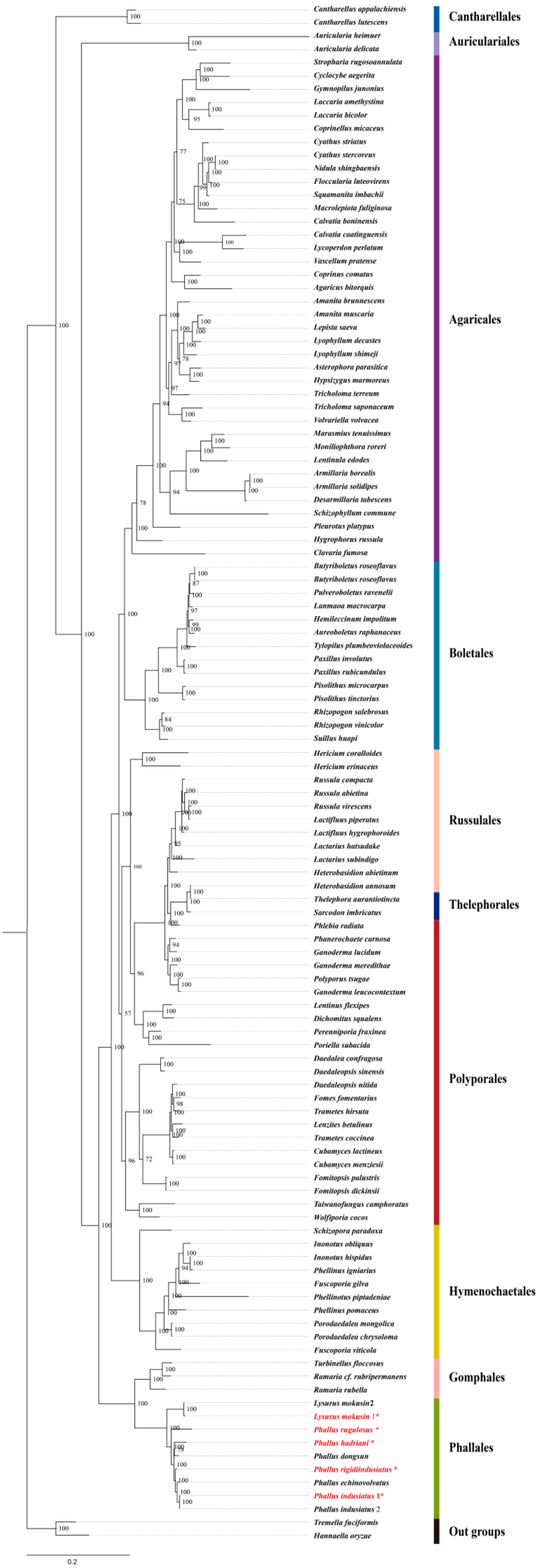

Figure S6. The phylogenetic tree of PCG12R dataset constructed using IQ-TREE.
